# Enhancing Security in Visible Light Communication: A Tabu-Search-Based Method for Transmitter Selection

**DOI:** 10.3390/s24061906

**Published:** 2024-03-16

**Authors:** Ge Shi, Wei Cheng, Xiang Gao, Fupeng Wei, Heng Zhang, Qingzheng Wang

**Affiliations:** 1 The School of Information Engineering, North China University of Water Resources and Electric Power, Zhengzhou 450046, China; 2 The School of Electronics and Information, Northwestern Polytechnical University, Xi’an 710129, China; pupil_119@nwpu.edu.cn (W.C.); 1486812648@mail.nwpu.edu.cn (X.G.);

**Keywords:** physical layer security, visible light communication, LED selection, secrecy rate

## Abstract

In this paper, we explore the secrecy performance of a visible light communication (VLC) system consisting of distributed light-emitting diodes (LEDs) and multiple users (UEs) randomly positioned within an indoor environment while considering the presence of an eavesdropper. To enhance the confidentiality of the system, we formulate a problem of maximizing the sum secrecy rate for UEs by searching for an optimal LED for each UE. Due to the non-convex and non-continuous nature of this security maximization problem, we propose an LED selection algorithm based on tabu search to avoid getting trapped in local optima and expedite the search process by managing trial vectors from previous iterations. Moreover, we introduce three LED selection strategies with a low computational complexity. The simulation results demonstrate that the proposed algorithm achieves a secrecy performance very close to the global optimal value, with a gap of less than 1%. Additionally, the proposed strategies exhibit a performance gap of 28% compared to the global optimal.

## 1. Introduction

Visible light communication (VLC) is an emerging wireless technology that uses visible light to transmit data. VLC works by modulating the intensity of optical signals from light sources to encode and transmit information. VLC has gained significant attention recently due to its potential for fast, secure, and energy-efficient data transmission [1]. It can operate where radio frequency (RF) signals are restricted, such as in hospitals and aircraft. VLC also offers transmission security since the signals do not penetrate walls and are not susceptible to electromagnetic interference. VLC has various applications, including indoor positioning [2], smart lighting, and high-speed internet access [3].

VLC systems have a broadcasting characteristic, which allows multiple users (UEs) to access the information transmitted from every light-emitting diode (LED) that is inside their detective areas. A malicious UE can potentially gather confidential data not intended for them. Traditional upper techniques may not effectively safeguard VLC systems since each transmission requires private key exchanges and management. Fortunately, physical layer security (PLS) has appeared as a promising method to enhance wireless security by utilizing wireless channel characteristics. In PLS, the performance is determined by the transmission performance gap between a legitimate channel and an eavesdropper (Eve) channel [4]. Therefore, to enhance the security of VLC systems, strategies must be designed to improve the signal-to-interference-plus-noise ratio (SINR) of the legitimate UE while degrading that of the Eve. This approach has been extensively investigated in RF systems, but PLS in VLC systems requires special investigation due to their differing characteristics.

Beamforming-based techniques demonstrate superior performance as they enable transmitters to steer the direction of optical signals and adjust their power levels [5,6]. By dynamically controlling these parameters, beamforming-based methods can focus the transmitted light toward specific UEs or areas of interest. This targeted transmission improves signal strength, enhances coverage, avoids eavesdropping, and reduces interference from other sources, leading to a superior performance in terms of signal reception and reliability. However, beamforming-based methods may require additional equipment, such as lenses and signal processing circuits, to adjust the amplitude and direction of the optical signals to create a beam targeting UEs. VLC systems are typically intensity-modulation/direct-detection systems, which use incoherent light and cannot directly control the phase of the light wave. Beamforming devices that are designed for coherent optical communication systems, such as those using infrared radiation lasers [7], cannot be directly transplanted into VLC systems.

Another promising technique for improving the performance of VLC systems is LED selection. While LED selection methods may not achieve the same level of performance as beamforming-based methods, they offer a compromise between performance and complexity [8]. The computational complexity comparison between these two methods can be analyzed based on two factors: the solution space and the problem dimension. Beamforming-based methods refine solutions in a continuous space, while LED selection methods search for solutions in a discrete space. Due to its finer-grained solution space, the former methods generally require more iterations compared to the latter methods. In addition, the problem dimension for beamforming-based methods is *M* times larger than that for LED selection methods, where *M* represents the number of UEs. In beamforming-based methods, the optimization variables for a secrecy-rate maximization problem are *M* beamforming vectors that allocate the coefficients from LEDs to each UE, with a size of 1×K, where *K* represents the number of LEDs. On the other hand, in LED selection methods, the optimization variable is one vector that includes the indices of LEDs, with a size of 1×K.

To enhance the transmission quality in LED selection methods, the optimal criterion is often to associate an LED–UE pair that has the shortest Euclidean distance. This is because the useful signal strength transmitted from the LED towards the UE is the strongest in this case. Fangxin et al. proposed an LED selection algorithm based on the support vector machine of a VLC system to improve the bit error rate, where the Euclidean distance are selected as the key metric of the system to construct the label vector of the training samples [9]. Yitian et al. designed an Euclidean distance-based LED selection scheme to improve the bit error rate by reducing the effect of channel correlation [10]. Yang et al. proposed a hybrid dimming scheme to maximize the throughput of a multiple-user, multiple-cell VLC system [11]. The LED selection subproblem was formulated as a mixed-integer problem and solved using a penalty method. For security purposes, the main idea behind LED selection is to choose the best combinations of LEDs and UEs that can maximize the data rates of UEs while minimizing that of Eves. Cho et al. proposed an LED selection scheme that chooses the nearest LED for UEs to reduce the secrecy outage probability of a VLC system, providing a practical and near-optimal solution [12]. Motivated by these results, a TS-based LED selection scheme is proposed to maximize the sum secrecy rate of VLC systems in this paper, which has not been investigated yet. A brief summary of the above work is given in Table 1.

In this paper, we investigate a multi-UE VLC channel that comprises multiple LEDs with the objective of transmitting confidential messages to UEs in the presence of an active Eve. Assuming that UEs and the active Eve send feedback through uplink media, the CSI of UEs and Eve is known by the center control unit. In the center control unit, a UE selects an LED based on an algorithm or strategy. The primary goal of the study is to improve the secrecy performance by assigning a distinct LED to each UE while taking the presence of an Eve into account. To achieve this goal, we formulate a sum secrecy rate maximization problem with integer variables, which is non-convex in nature, and introduce a tabu search (TS)-based algorithm that provides a sub-optimal solution with low complexity. To explore the solution spaces in this algorithm, we design a neighboring vector whose element selects a random LED within the UE’s receiving area. Furthermore, we design three simple LED selection strategies to reduce the complexity further. Simulation results and comparisons of the proposed algorithm with three simple strategies and global search are provided to demonstrate the effectiveness of the proposed algorithm.

The rest of the paper is organized as follows: In Section 2, we describe the system model for the downlink transmission of the multi-UE VLC system. Section 3 presents the TS-based LED selection algorithm and three strategies while providing a complexity and convergence analysis. In Section 4, we offer numerical simulation results and comparisons. Finally, we conclude the paper in Section 5.

## 2. System Model

There are *M* UEs and *K* LEDs in an interior setting with the presence of an Eve. We assume that a UE selects one LED to connect, and the UE-to-LED connection is a one-to-one pair. $x=\left[k_{1},k_{2},k_{m},\cdots ,k_{M}\right]$ is an index vector that indicates the connection between LEDs and UEs, where $k_{m}\in A$ is the index of the LED that is associated with the *m*-th UE, and A={1,2,k,⋯,K} denotes a set that contains the index of every LED. Once the index vector x is determined, each UE receives its confidential signal from a specific LED. LEDs that are not selected by any UE are only used for illumination, not for transmitting signals. However, due to the broadcasting nature of VLC, a UE also receives signals from nearby LEDs which are selected by other UEs.

**Figure 1:**
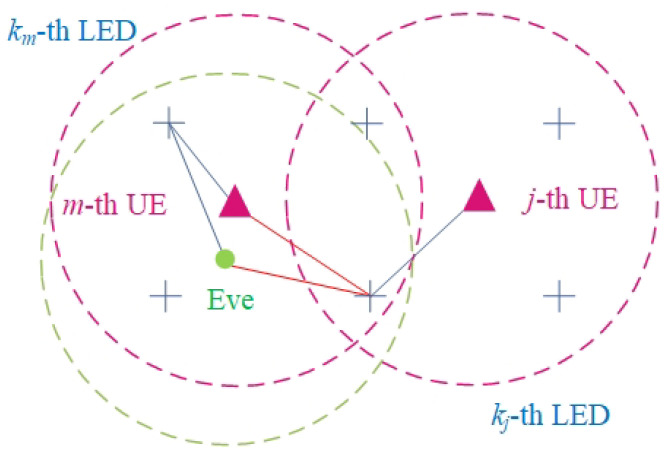
System model.

For example, as shown in Figure 1, the *m*-th UE (magenta triangle) selects the $k_{m}$-th LED (blue fork) and the *j*-th UE selects the $k_{j}$-th LED. The *m*-th UE receives two different signals in its reception range. The reception ranges of UEs are denoted as magenta dashed circles. The signal from the $k_{m}$-th LED is confidential information, whereas the signal from the $k_{j}$-th LED is considered user interference. The useful links and interference links are represented by blue lines and red lines, respectively. When an Eve (green solid circle) intends to eavesdrop on the *m*-th UE, the signal from the $k_{j}$-th LED is regarded as interference. The reception ranges of the Eve are denoted as a green dashed circle. In the meantime, the *j*-th UE only receives its signal from the $k_{j}$-th LED since no LED selected by other UEs is within its reception range.

In the general case, assuming the index vector x is fixed, the signal received at the *m*-th UE is formulated as follows:

(1) $$y_{m}=h_{k_{m},m}s_{m}+\sum_{j=1,\;j\neq m}^{M}h_{k_{j},m}s_{j}+n_{m},$$

where $E\left\{s_{m}^{2}\right\}=E\left\{s_{j}^{2}\right\}=1$ and $E\left\{s_{m}\right\}=E\left\{s_{j}\right\}=0$. The channel gain from the $k_{m}$-th LED to the *m*-th UE is represented as $h_{k_{m},m}$; $s_{m}$ is the information signal for the *m*-th UE; the channel gain from the $k_{j}$-th LED to the *m*-th UE is $h_{k_{j},m}$; and $s_{j}$ is the information signal for the *j*-th UE and part of the interference signals for the *m*-th UE. If the $k_{j}$-th LED falls outside the *m*-th UE’s reception range, the channel gain is zero, i.e., $h_{k_{j},m}=0$. The noise source inside the receivers’ circuit is mainly dominated by thermal noise and shot noise. This is modeled as additive zero-mean Gaussian noise, i.e., $n_{m}∼N\left(0,\xi^{2}\right)$. The channel gain from the $k_{m}$-th LED to the *m*-th UE can be expressed as [13]:

(2) $$h_{k_{m},m}=\frac{\left(\gamma +1\right)A_{R}}{2\pi d_{k_{m},m}^{2}}\cos{\left(\phi_{k_{m},m}\right)}^{\gamma }\cos\left(\varphi_{k_{m},m}\right)f\left(\varphi_{k_{m},m}\right)g_{\mathrm{of}},$$

where $\gamma =\frac{-1}{\log_{2}\cos\phi_{1/2}}$ denotes the Lambertian emission order, $\phi_{1/2}$ and $A_{R}$ are the half-intensity radiation angle and the area of receiver’s photodiode (PD), respectively, $d_{k_{m},m}$ is the Euclidean distance from the $k_{m}$-th LED to the *m*-th UE, $\phi_{k_{m},m}$ and $\varphi_{k_{m},m}$ are the irradiance angle and incidence angle, respectively, $g_{\mathrm{of}}$ is the gain of the optical filter, and $f(\varphi_{k_{m},m})$ denotes the gain of the optical concentrator, which is given by [13]:

(3) $$f\left(\varphi_{k_{m},m}\right)=\left\{\begin{array}{cc} \frac{q^{2}}{\sin^{2}\left(\varphi_{k_{m},m}\right)} & 0\leq \varphi_{k_{m},m}\leq \Theta ,\\ 0 & \varphi_{k_{m},m}\geq \Theta , \end{array}\right.$$

where *q* and Θ are the refractive index and the field of view (FoV) of the PD used at the *m*-th UE’s side, respectively. The extent of the FoV dictates the reception range for stationary UEs. The SINR of the *m*-th UE is calculated as follows:

(4) $$R_{m}=\frac{1}{2}\log_{2}\left(1+\frac{e}{2\pi }\frac{{\left|h_{k_{m},m}\right|}^{2}}{\sum_{j=1,\;j\neq m}^{M}{\left|h_{k_{j},m}\right|}^{2}+\xi^{2}}\right).$$

Assuming that the Eve overhears the confidential signal for the *m*-th UE, which is transferred from the $k_{m}$-th LED, the signal received by the Eve is formulated as:

(5) $$\begin{array}{c} \mathrm{ye}=g_{k_{m}}s_{m}+\sum_{j=1,\;j\neq m}^{M}g_{k_{j}}s_{j}+n_{e}, \end{array}$$

where $g_{k_{m}}$ is the channel gain from the $k_{m}$-th LED to the Eve; $s_{m}$ is the confidential signal for the *m*-th UE and also useful signal for the Eve; $g_{k_{j}}$ is the channel gain from the $k_{j}$-th LED to the Eve; and $s_{j}$ is the confidential signal for the *j*-th UE and part of interference signal for the Eve. Thus, the SINR of the Eve is calculated as follows:

(6) Re=12log21+e2πgkm2∑j=1,j≠mMgkj2+ξ2.

Based on the lower bound of the channel capacity considered in [14], the achievable secrecy rate of the *m*-th UE is given by substituting (4) and (6) into the following equations:

(7) SRm=[Rm−Re]+.

The sum secrecy rate maximization problem can be modeled as

(8) $$\;\max\sum_{m=1}^{M}{\mathrm{SR}}_{m},$$

(9) $$s.t.\;x=\left[k_{1},k_{2},k_{m},\cdots ,k_{M}\right]\in N^{M},k_{m}\in A,\;\;\forall m,$$

The above problem is an integer programming problem, and it involves searching for an optimal index vector x. The discrete nature of the solution space and the non-convexity of the problem are two of the main challenges in solving this problem. The former makes it impossible to find the optimal solution through simple interpolation or approximation, while the latter means there are many local optimal solutions, making it challenging to find the global optimal solution.

## 3. Proposed Algorithm and Strategies

### 3.1. Tabu Search-Based LED Selection Algorithm

LED selection can involve large search spaces due to the number of possible combinations of LEDs and UEs. Decision tree algorithms explore multiple branches and evaluate potential splits at each level, which can lead to an increased computational complexity and longer processing times [15]. Genetic algorithms, with their population-based approach and genetic operations, may have a higher computational complexity as they evaluate and evolve multiple solutions in parallel [16]. However, tabu search’s local search approach and memory-based mechanisms can efficiently explore the neighborhood and avoid revisiting local optima, making it suitable for handling large search spaces [17]. The algorithm was first introduced in [18] and has since been extensively studied and applied in various fields [19,20]. Whei-Min developed an improved TS algorithm for a nonlinear economic dispatch problem that classic Lagrange-based algorithms failed to solve [19]. Srinidhi proposed a layered detection approach in conjunction with TS and showed that it works very well in terms of both performance and complexity in multiple-input, multiple-output systems with a large number of antennas [20].

The TS algorithm begins with an initial solution, defines a set of neighboring solutions based on a neighborhood criterion, and moves to the best one among the neighboring solutions. This process has a certain number of iterations, after which the algorithm is stopped and the best solution of all the iterations is declared as the final result. In defining the neighborhood of the solution in a given iteration, the algorithm attempts to avoid cycling by prohibiting the moves to the solutions of the past few iterations. The solutions of the past few iterations are recorded in the ‘tabu list’ and parameterized by the ‘tabu period’, which is changed depending on the number of repetitions of the solutions that are marked in the search path. The performance of TS is highly dependent on the selection of its parameters, such as definitions of the neighborhood, the tabu list, and the tabu period.

Neighborhood Definition: Let $x^{\left(l\right)}=\left[k_{1}^{\left(l\right)},k_{2}^{\left(l\right)},\cdots ,k_{M}^{\left(l\right)}\right]$ denote an index vector belonging to the solution space in the *l*-th iteration, where $k_{m}\in A$ is the index of the LED that is selected by the *m*-th UE. The neighborhood of the $k_{m}$-th LED for the *m*-th UE is chosen based on $B_{m}$. $B_{m}$ is a fixed subset of A, and it contains the indices of every reachable LED for the *m*-th UE. As long as the *m*-th UE does not move, $B_{m}$ is a fixed subset. The cardinality and members of this set are not the same for different UEs, but depend on their FoV and position. Note that the maximum and minimum values of the cardinality are *K* and 1, respectively. We refer to a neighbor vector $z^{\left(l\right)}\left(q\right)=\left[z_{1}^{\left(l\right)}\left(q\right),\;z_{2}^{\left(l\right)}\left(q\right),\cdots ,z_{M}^{\left(l\right)}\left(q\right)\right]$ as the *q*-th neighbor of $x^{\left(l\right)}$, where the *m*-th element $z_{m}^{\left(l\right)}\left(q\right)$ is the *q*-th neighbor of $k_{m}^{\left(l\right)}$, q=1⋯M. The $z_{m}^{\left(l\right)}\left(q\right)$ -th LED is the *q*-th neighbor of the $k_{m}^{\left(l\right)}$-th LED for the *m*-th UE, the formula of which is written as follows:

(10) $$z_{m}^{\left(l\right)}\left(q\right)=\omega \left(k_{m}^{\left(l\right)}\right),\forall q=1\cdots M.$$

where $\omega (k_{m}^{\left(l\right)})$ represents that $z_{m}^{\left(l\right)}\left(q\right)$ is randomly selected from $B_{m}$ but does not include $k_{m}^{\left(l\right)}$, i.e., $z_{m}^{\left(l\right)}\left(q\right)\in B_{m}\backslash k_{m}^{\left(l\right)}$. We choose *M* neighbor vectors that differ from a given vector $x^{\left(l\right)}$ in the solution space. An operation on $x^{\left(l\right)}$ which gives $x^{\left(l+1\right)}$ belonging to the neighborhood of $x^{\left(l\right)}$ is referred to as a move. The algorithm is said to execute a move if $x^{\left(l+1\right)}=z^{\left(l\right)}\left(q\right)$. We note that the number of candidates to be considered for a move is equal to *M* in any of the iterations.

Tabu List: A tabu list T of size M×K is a matrix whose entries denote the tabu values of moves. The tabu value of a move means that the move cannot be considered for that many successive iterations. There are *M* rows in T, where each row represents the indices of UEs from 1 to *M*. The *K* columns of the T matrix correspond to the indices of LEDs from 1 to *K*. In other words, the $\left(m,k_{m}\right)$-th entry of the tabu list corresponds to the connection between the $k_{m}$-th LED and the *m*-th UE. The entries of the tabu list are updated in each iteration, and they are used to determine the direction in which the search proceeds.

Tabu Period: The tabu period, a non-negative integer parameter, is defined as follows. If combinations of LEDs and UEs are accepted as the next move in an iteration, these combinations will be restricted for several subsequent iterations. The number of restricted iterations is denoted by the tabu period.

TS Algorithm: Let $\alpha^{\left(l\right)}$ be a solution that has the best sum secrecy rate found till the *l*-th iteration. The algorithm starts with an initial solution vector $x^{\left(0\right)}$. Set $\alpha^{\left(0\right)}=x^{\left(0\right)}$. Note that different initial solutions can lead to different results. All the entries of the tabu list are set to zero. The following steps (1) to (3) are performed in each iteration. Consider the *l*-th iteration and the current solution $x^{\left(l\right)}$ in the algorithm, l≥0.

Step (1): The sum secrecy rate of the neighbor vector $\mathrm{SR}(z^{\left(l\right)}\left(q\right))$ is computed. Let

(11) $$z^{\left(l\right)}\left(q^{*}\right)=\arg\max_{q}\mathrm{SR}\left(z^{\left(l\right)}\left(q\right)\right),\forall q=1\cdots M.$$

The neighbor vector $z^{\left(l\right)}\left(q^{*}\right)$ is accepted as the next move if any one of the following two conditions is satisfied:

(12) $$\;\mathrm{SR}\left(z^{\left(l\right)}\left(q^{*}\right)\right)>\mathrm{SR}\left(\alpha^{\left(l\right)}\right),$$

(13) $$\;T(m,{z_{m}}^{\left(l\right)}\left(q^{*}\right))=0,\forall m=1\cdots M.$$

The first equation indicates that the secrecy performance of the neighbor vector $z^{\left(l\right)}\left(q^{*}\right)$ surpasses that of the best index vector $\alpha^{\left(l\right)}$ up till the *l*-th iteration. The second formula denotes that the $\left(m,{z_{m}}^{\left(l\right)}\left(q^{*}\right)\right)$-th entry in the tabu list is marked as zero, which means that the connection between the ${z_{m}}^{\left(l\right)}\left(q^{*}\right)$-th LED and the *m*-th UE is not forbidden. If this neighbor is not accepted as the next move (i.e., either one of the conditions above is not satisfied), the neighbor vector $z^{\left(l\right)}\left(q^{**}\right)$ with the second best sum secrecy rate is found such that

(14) $$z^{\left(l\right)}\left(q^{**}\right)=\arg\max_{q:q^{**}\neq q^{*}}\mathrm{SR}\left(z^{\left(l\right)}\left(q\right)\right).$$

Next, check for acceptance of $z^{\left(l\right)}\left(q^{**}\right)$ by substituting it in (12) and (13). If this also cannot be accepted, the procedure is repeated for the neighbor vector $z^{\left(l\right)}\left(q^{***}\right)$ with the third-best sum secrecy rate, and so on. If any unrestricted neighbor is accepted as the next move, proceed to Step (2). Otherwise, advance to Step (4). Continue this process until an acceptable move is made.

Step (2): Let $z^{\left(l\right)}\left(\hat{q}\right)$ be a neighbor vector for which the move is permitted. Make

(15) $$x^{\left(l+1\right)}=z^{\left(l\right)}\left(\hat{q}\right),$$

where the *m*-th element ${z_{m}}^{\left(l\right)}\left(\hat{q}\right)$ of the neighbor vector $z^{\left(l\right)}\left(\hat{q}\right)$ represents the index of the $\hat{q}$-th neighbor LED for the $k_{m}$-th LED. It should be noted that the best permissible neighbor vector is selected as the solution vector for the subsequent iteration. Next, if this neighbor vector satisfies the first condition (12), it is assigned to the best solution $\alpha^{\left(l+1\right)}$ in the (l+1)-th iteration.

Step (3): The connection between the ${z_{m}}^{\left(l\right)}\left(\hat{q}\right)$-th LED and the *m*-th UE should be prohibited for several upcoming iterations to prevent cycling. Thus, the $\left(m,{z_{m}}^{\left(l\right)}\left(\hat{q}\right)\right)$-entry of the tabu list is assigned by the tabu period, denoted as *M*:

(16) $$\;\;T(m,{z_{m}}^{\left(l\right)}\left(\hat{q}\right))=M,\;\forall m=1\cdots M,$$

Step (4): All the non-zero entries of the tabu list are reduced by one, which is achieved as follows:

(17) $$T\left(m,k_{m}\right)=\max\left\{T\left(m,k_{m}\right)-1,0\right\},\;\forall k_{m}=1\cdots K,$$

The algorithm terminates in Step 4 if the following stopping criterion is satisfied; otherwise, it goes back to Step (1).

**Figure 2:**
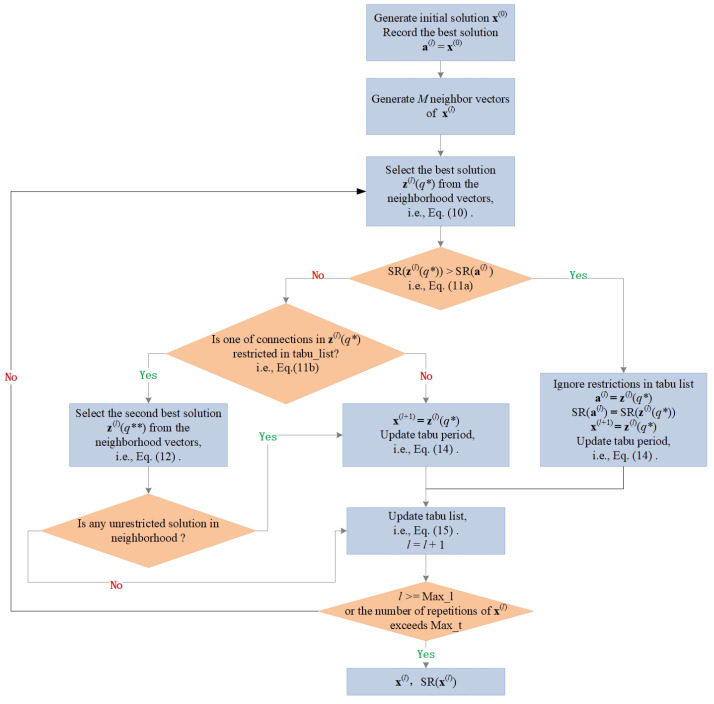
The procedure of the proposed algorithm (Orange diamond and blue rectangle are depicted for the decision and process in the algorithm, respectively).

Stopping Criterion: The algorithm described above is stopped if the maximum number of iterations Max_l is reached. Also, if the total number of repetitions of the current solution is greater than a threshold Max_t, the algorithm is stopped. The average complexity of the algorithm is O(MK). The procedure of the algorithm is further illustrated in Figure 2.

### 3.2. Simple LED Selection Strategies

Three simple LED selection strategies are proposed as benchmarks in this scenario, which require no iterations and have a lower complexity.

Random strategy: In this strategy, each UE is assigned to a random LED within its reception range. The solution vector, denoted as $x=\left[k_{1},k_{2},k_{m},\cdots ,k_{M}\right]$, represents the assignment of LEDs to UEs, where $k_{m}$ is the index of the LED that the *m*-th UE randomly chooses from the set $B_{m}$. Note that the random strategy does not consider any optimization criteria or system constraints. If any LED is already connected to a UE, then it cannot be selected as the transmitter for other UEs.

Channel gain-based strategy: In this strategy, each UE is assigned to the LED with the highest channel gain, which is similar to the LED selection method proposed in [12]. $k_{m}\in B_{m}$ is the index of the LED that is associated with the *m*-th UE.

Eve’s channel gain-based strategy: In this strategy, each UE is allocated to the LED with the highest channel gain that is greater than Eve’s channel gain. If a UE’s channel gain is less than Eve’s, the corresponding LED is banned from being selected by that UE. Thus, the indices of the banned LEDs are eliminated from the set $B_{m}$. Assuming that β is the set containing the indices of banned LEDs, $k_{m}\in B_{m}\backslash \beta$ is the index of the LED that is associated with the *m*-th UE.

Since the main procedure of the above strategies involves sorting channel gains, the average complexity of the above three strategies is O(K).

### 3.3. Global Search

The result of the global search is used to measure the optimality of the proposed algorithm and strategies. To achieve this, a list that contains all possible solutions is created, and the best solution is traversed by substituting every solution vector $x=\left[k_{1},k_{2},k_{m},\cdots ,k_{M}\right]$ into (8). Thus, the average complexity of the algorithm is $O(K^{M})$.

### 3.4. Convergence Analysis

**Figure 3:**
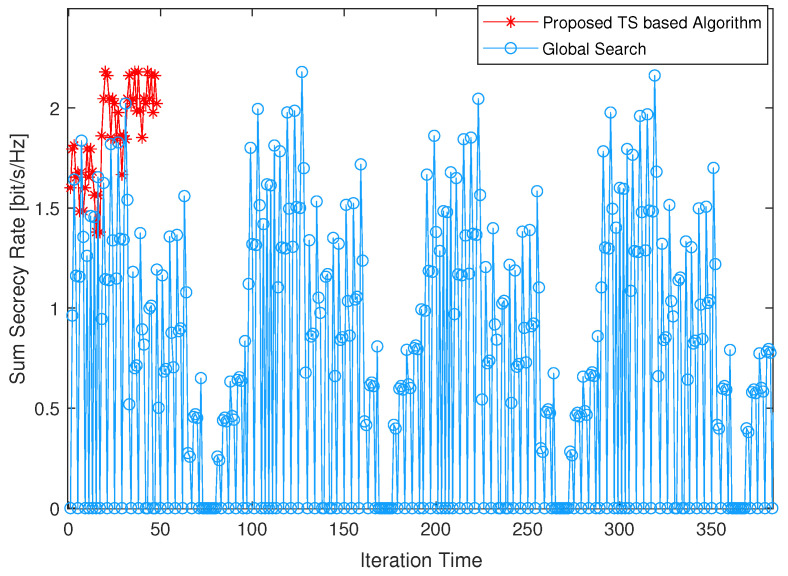
Comparison of the proposed algorithm and global search regarding convergence performance.

Figure 3 illustrates the iteration times of the proposed algorithm in comparison to global search. The proposed algorithm and global search achieve the same optimal value of 2.18 bit/s. The global search terminated at the 384th iteration. The optimal value was determined after an ergodic examination of all reasonable combinations between 5 UEs and 25 LEDs. Two kinds of scenarios need to be excluded from the examination: multiple UEs connecting to the same LED, and one LED connecting to one UE not inside its illumination area. In comparison, the proposed algorithm terminated at the 50th iteration when it reached the stopping criterion (that is, the repetition number of the best solution is greater than the threshold). As shown in Figure 3, the algorithm reached the best value four times. The best solution was first obtained in the 21st iteration, and it was also the optimal value.

## 4. Numerical Results

**Figure 4:**
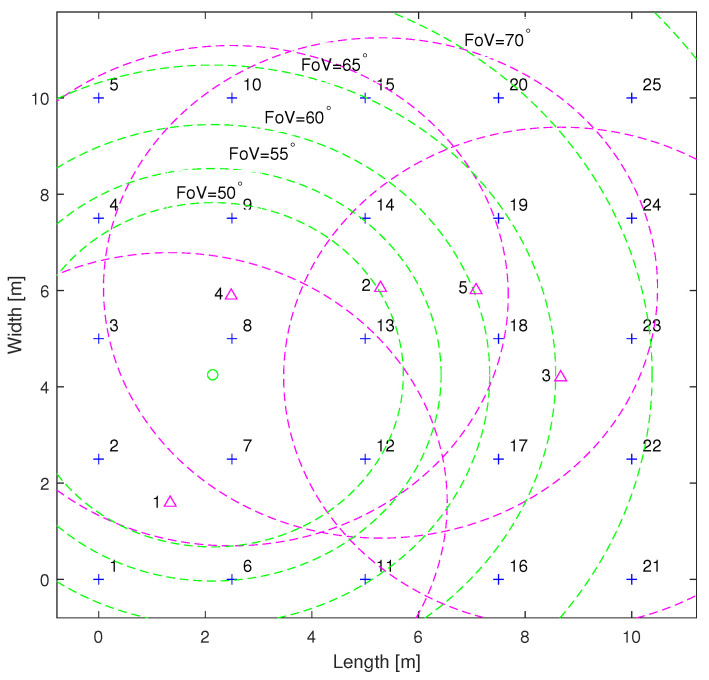
Placement of LEDs (blue) and example of a layout for UEs (magenta) and an Eve (green).

In this section, we study the secrecy performance of the proposed VLC system in a 10m×10m×3m indoor setting. Figure 4 demonstrates the geometrical configuration of 25 LEDs within an indoor room. Note that the simulations below employ a Monte Carlo-based approach, and the secrecy performance is determined by averaging the results from 500 different instances. In each instance, the coordinates of the UEs and the Eve are randomly generated using a uniform distribution within the interval of [0,10]. The parameters used in the following simulations are shown in Table 2.

Figure 4 shows a layout example of five UEs and one Eve. In this case, the coordinates of the UEs are (1.34, 1.59, 0.8), (5.28, 6.05, 0.8), (8.66, 4.19, 0.8), (2.48, 5.89, 0.8), and (7.07, 6.00, 0.8), while the coordinates of the Eve are (2.13, 4.25, 0.8). Blue forks denote the coordinates of the LEDs. The positions of the UEs are marked with magenta triangles, and magenta dashed circles denote their respective coverage areas. The green circle represents Eve’s location, and the green dashed circles denote its coverage areas.

**Figure 5:**
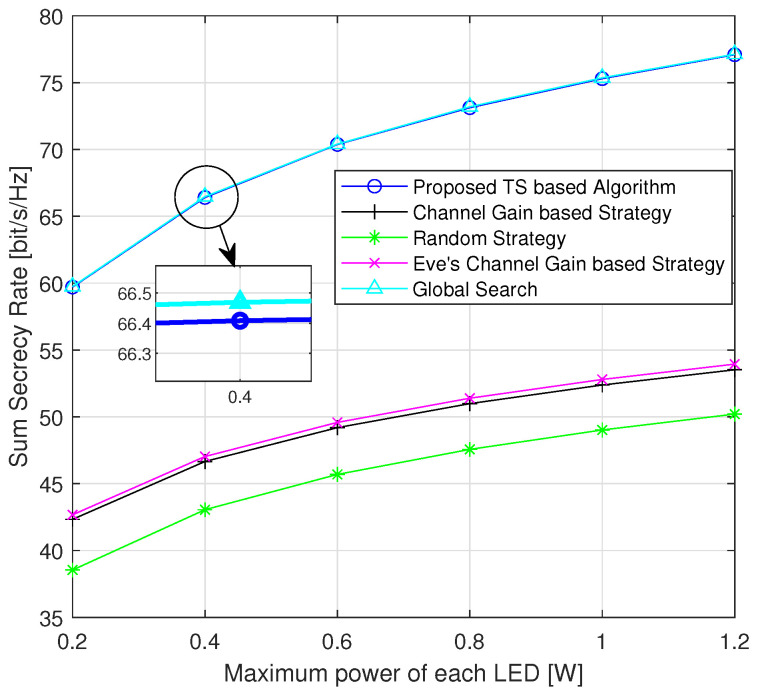
Average sum secrecy rate with respect to the maximum power of each LED (randomly generated positions of UEs and Eves for each instance).

Figure 5 displays the changes in the sum secrecy rate as a function of the maximum power of each LED. The improvement in the secrecy rate is attributed to the increased signal strength transmitted by the LED. When the maximum power of the LED increases, the *m*-th UE can receive a stronger confidential signal from its intended LED. If there is no interference signal from nearby LEDs, the increased power directly contributes to an enhancement in the achievable data rate at the *m*-th UE. Furthermore, the results of the global search represent the optimal performance in this scenario, and the proposed algorithm’s performance is very close to it. The proposed algorithm outperforms three simple strategies, and the performance gap between them becomes more significant with increasing power. Notably, the performance of the Eve’s channel-gain-based strategy is superior to that of the channel-gain-based strategy. This is because the latter does not take into account the position of the Eve, whereas the former allows UEs to reject the connection of LEDs that provide a better channel quality to the Eve.

**Figure 6:**
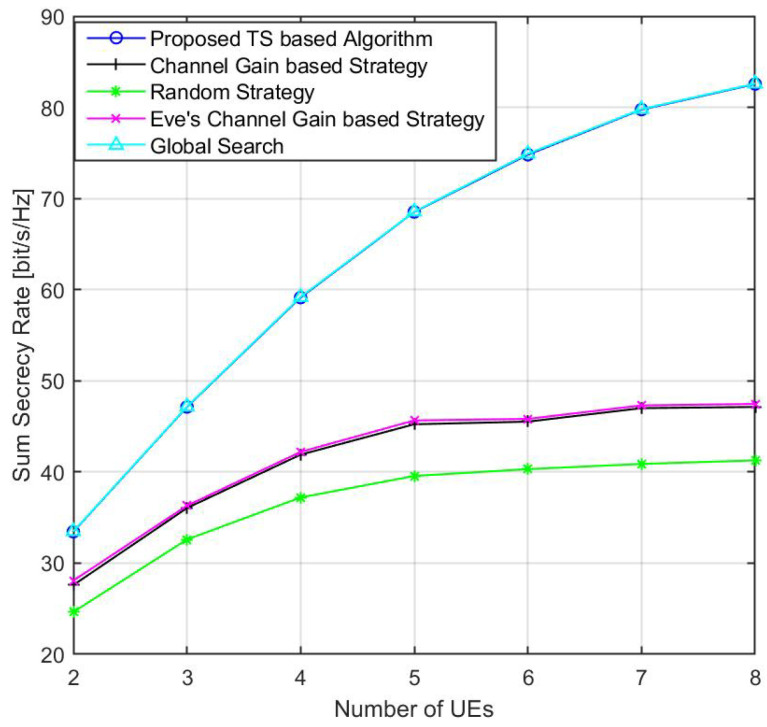
Average sum secrecy rate with respect to the total number of randomly placed UEs.

Figure 6 illustrates the variation in the sum secrecy rate with respect to the number of UEs. As the number of UEs increases, the sum secrecy rate initially experiences a rapid growth. This is due to the increased potential for multi-user diversity. UEs that are in favorable positions, for instance, those far away from the Eve, contribute to a higher sum secrecy rate. However, the growth rate slows down as the number of UEs continues to increase. This is attributed to the increased inter-user interference and the limited number of LEDs that can be selected for UEs.

**Figure 7:**
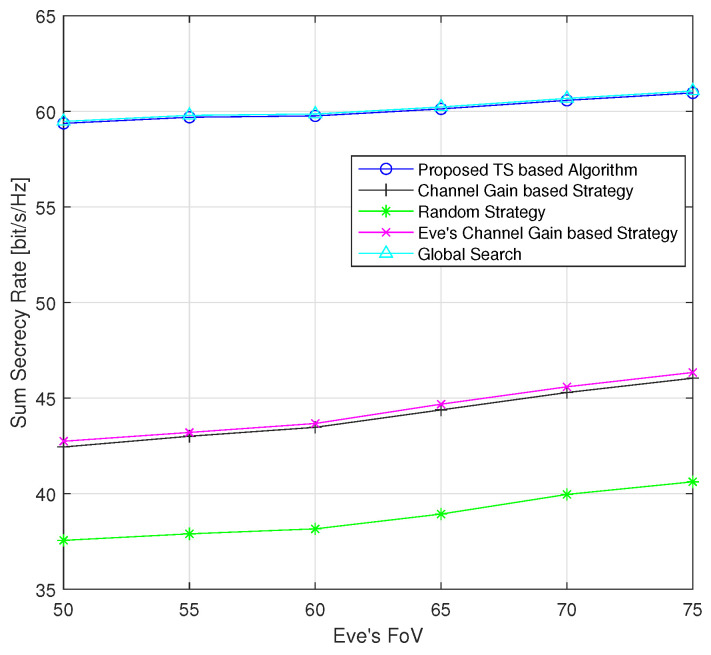
Average sum secrecy rate with respect to the Eve’s FoV (randomly generated positions of UEs and Eves for each instance).

Figure 7 illustrates the performance of the sum secrecy rate as a function of the Eve’s FoV. The FoV of the UEs is 50 degrees. The secrecy performance exhibits a slightly increasing trend with an increasing FoV. This is because both interference and useful signals grow on the Eve’s side. As depicted in Figure 4, the green circles represent the detectable areas of the Eve. As the FoV increases, the green circles become larger, encompassing more UEs and LEDs, which allows the Eve to receive useful signals from more UEs. For example, at a FoV of 70 degrees, the Eve begins to receive signals from the third UE. Therefore, compared with the case where its FoV is smaller than 70 degrees, the Eve’s achievable rate becomes greater than zero when overhearing the third UE. However, if the Eve overhears other UEs, their achievable rates become lower due to interference from the third UE.

**Figure 8:**
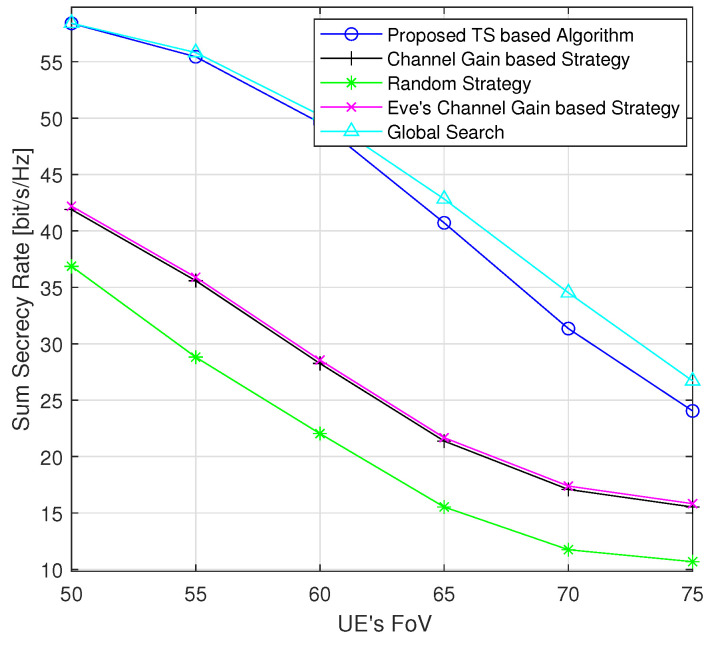
Average sum secrecy rate with respect to the UEs’ FoVs (randomly generated positions of UEs and Eves for each instance).

Figure 8 displays the changes in the sum secrecy rate as a function of the FoV of the UEs, while the Eve has a fixed FoV of 50 degrees. As the FoV of the UEs increases, the downward trend in the secrecy performance becomes less pronounced. This is because with a wider FoV, UEs detect signals from more LEDs and receive a stronger interference signals. Once the FoV becomes large enough to connect all LEDs, the interference remains constant and hence the secrecy performance stabilizes.

**Figure 9:**
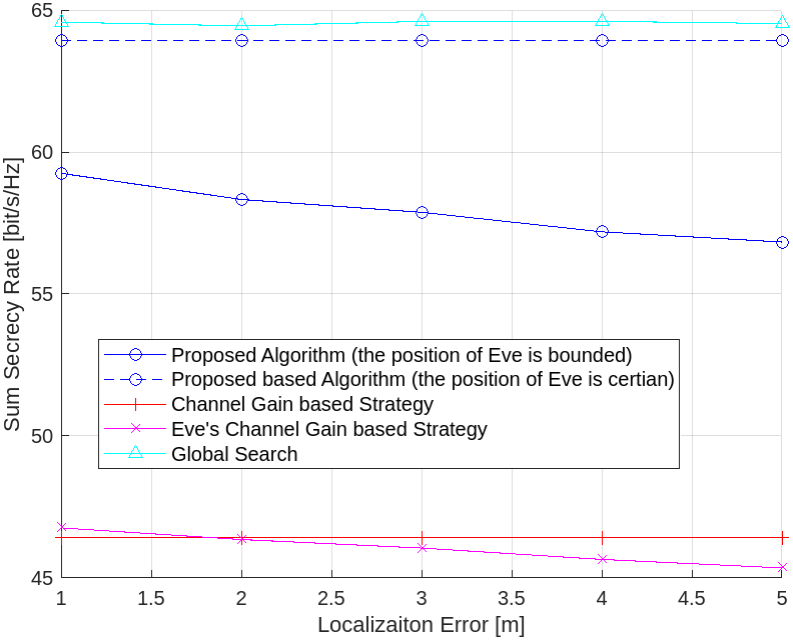
Average sum secrecy rate with respect to the localization error of the Eve (randomly generated positions of UEs and Eves for each instance).

Figure 9 shows that the accuracy of the Eve’s position influences the performance of the proposed algorithm. Both the secrecy performance of the proposed algorithm and the proposed Eve’s channel-gain-based strategy gradually deteriorate as the localization error of the Eve increases. However, the performance of the channel-gain-based strategy remains stable, as this strategy assigns the LEDs to the UEs without considering the position of the Eve.

## 5. Conclusions

In this paper, we present a multi-LED VLC system incorporating multiple UEs and a single Eve. Depending on the perfect CSI of the UEs and the Eve, we propose an LED selection algorithm based on a TS to enhance the secrecy performance of the system. The simulation results demonstrate that the proposed algorithm offers a near-optimal solution, with a performance gap of less than 1%, while it achieves faster convergence in comparison to the global search. Additionally, we design three simple LED selection strategies to reduce the computational complexity, which all exhibit a performance gap of around 28% compared to the global search. The Euclidean distance-based criterion studied in previous work [12] has the same performance as the proposed channel-gain-based strategy (since in VLC, the channel gain is proportional to the distance). It is easy to implement in practical experiments since the distances between LEDs and the UE can be measured by using the received signal strength technique. However, the proposed algorithm achieves a near-optimal performance with a slight increase in computational complexity. With more hardware resources, such as adding more RAM to store the tabu list and using a camera to detect the position of the Eve, the proposed algorithm should be a better choice to improve the secrecy performance in a VLC system. Furthermore, the simulation results show the impact of various system parameters on the secrecy performance. The results indicate that increasing the number of UEs and the maximum power of each LED and reducing the UEs’ FoV improves the secrecy performance. On the other hand, augmenting the FoV of the Eve has a negligible impact on the secrecy performance.

## Figures and Tables

**Table 1:** Main features of related works and comparison with this paper.

Paper	Method	Main Contribution
Fangxin et al. [9]	Support vector machine	Improved bit error rate
Yang et al. [10]	Penalty method	Improved throughput
Yitian et al. [11]	Euclidean distance-based grouping strategy	Improved bit error rate
Cho et al. [12]	Euclidean distance-based strategy	Reduced secrecy outage probability
This paper	Tabu search	Improved secrecy rate

**Table 2:** Simulation parameters.

Parameter	Value
Room size (W×L×H)	$\left(10\times 10\times 4\right)\;m^{3}$
Average electrical ambient noise (ξ)	−98 dBm
Lambertian emission order (β)	1
Half-intensity radiation angle $\left(\theta_{1/2}\right)$	60°
PD surface area $\left(A_{R}\right)$	1 ${\mathrm{cm}}^{2}$
Optical filter gain $\left(g_{of}\right)$	1
Maximum power of an LED	23 dBm
PD FoV (Θ)	50°
Refractive index (*q*)	1.5

## Data Availability

Data are contained within the article.
